# Loss of the dermis zinc transporter ZIP13 promotes the mildness of fibrosarcoma by inhibiting autophagy

**DOI:** 10.1038/s41598-019-51438-9

**Published:** 2019-10-21

**Authors:** Mi-Gi Lee, Min-Ah Choi, Sehyun Chae, Mi-Ae Kang, Hantae Jo, Jin-myoung Baek, Kyu-Ree In, Hyein Park, Hyojin Heo, Dongmin Jang, Sofia Brito, Sung Tae Kim, Dae-Ok Kim, Jong-Soo Lee, Jae-Ryong Kim, Bum-Ho Bin

**Affiliations:** 1 Department of Food Science and Technology, College of Life Sciences, Kyung Hee University, Yongin, 446-701 Republic of Korea; 2 Department of Biochemistry and Molecular Biology, Yeungnam University College of Medicine, Daegu, 42415 Republic of Korea; 3 Korea Brain Bank, Korean Brain Research Institute, Daegu, 41062 Republic of Korea; 4 Department of Bological Sciences, Ajou University, Suwon, 443-380 Korea; 5 Department of Biomedical Sciences, Major in Molecular Medicine, Ajou University Graduate School, Suwon, South Korea; 6 Department of Pharmaceutical Engineering, Inje University, Gimhae-si, 50834 Korea; 7 Graduate School of Biotechnology, Kyung Hee University, Yongin, 446-701 Republic of Korea

**Keywords:** Cancer metabolism, Sarcoma

## Abstract

Fibrosarcoma is a skin tumor that is frequently observed in humans, dogs, and cats. Despite unsightly appearance, studies on fibrosarcoma have not significantly progressed, due to a relatively mild tumor severity and a lower incidence than that of other epithelial tumors. Here, we focused on the role of a recently-found dermis zinc transporter, ZIP13, in fibrosarcoma progression. We generated two transformed cell lines from wild-type and ZIP13-KO mice-derived dermal fibroblasts by stably expressing the Simian Virus (SV) 40-T antigen. The ZIP13^−/−^ cell line exhibited an impairment in autophagy, followed by hypersensitivity to nutrient deficiency. The autophagy impairment in the ZIP13^−/−^ cell line was due to the low expression of LC3 gene and protein, and was restored by the DNA demethylating agent, 5-aza-2’-deoxycytidine (5-aza) treatment. Moreover, the DNA methyltransferase activity was significantly increased in the ZIP13^−/−^ cell line, indicating the disturbance of epigenetic regulations. Autophagy inhibitors effectively inhibited the growth of fibrosarcoma with relatively minor damages to normal cells in xenograft assay. Our data show that proper control over autophagy and zinc homeostasis could allow for the development of a new therapeutic strategy to treat fibrosarcoma.

## Introduction

Four types of cancers have been classified based on the cell type from which they initiate: blood cancer, germinoma (from germ line), carcinoma (from epithelial cells), and non-hematopoietic mesenchymal cancer, called sarcoma^1^. Blood cancers are divided into leukemia and lymphoma, depending if they mature in the bloodstream or the lymphatic system. The most common types of sarcoma develop from connective tissues such as bones and soft tissues^1^. Soft tissue sarcomas may start in dermis, muscle, nerves, and fat, and are found in various parts of the body including limbs, head, face, neck, and trunk. Fibrosarcoma, one of the most common sarcomas found in humans and animals, is derived from fibrous tissue and is associated with the presence of immature proliferating fibroblasts^1^. Fibrosarcoma is defined as a ‘malignant neoplasm composed of fibroblasts with variable collagen production’ by the World Health Organization^2^. In humans, adult- and infantile-type fibrosarcomas are mainly distinguished by their initial incident ages^2^. Infantile fibrosarcoma represents approximately 5% to 10% of all sarcomas in infants below 1 year of age, and exhibit a good prognosis: over 80% of patients are cured. Adult fibrosarcoma occurs in middle-aged and older adults, and recent data revealed a higher incidence in males^2^. However, fibrosarcoma has not been studied extensively, since it is a rare and mild tumor compared to other common epithelial cancers.

Recent studies revealed that, in cancer cells, autophagy displays different characteristics depending on cancer origin and cell type, drawing considerable attention in cancer research^3,4^. Autophagy is a eukaryotic cell-conserved degradative process activated in response to nutrient-deficiency or other stress conditions^5^. Autophagy generally consists of 4 steps^5^. The autophagic process is initiated by the appearance of preautophagosomal structures with a double membrane deriving from intracellular compartments or de novo lipid synthesis^5^. During this step, microtubule-associated protein light chain 3 (LC3), the most well-known autophagy-associated protein, is processed. Newly translated cytosolic LC3 is immediately and proteolytically cleaved by ATG4, a cysteine protease, at the C-terminus, forming LC3-I^5–7^. Autophagy induction triggers LC3-I conversion to LC3-II by conjugating phosphatidylethanolamine (PE) to the carboxyl-terminal glycine of LC3-I via the E1 and E2 proteins in the ubiquitin-like system. LC3-II is then recruited and integrated into the growing phagophore to play a role in membrane fusion^5,8^. The second step is the capturing of cellular organelles and proteins within a double-membrane vesicle (300–900 nm) called the autophagosome^5^. LC3-II is present on the internal and external surfaces of the autophagosome to promote its formation and to select the cargo for degradation^8^. The third step is the docking and fusion of the autophagosome with late endosomes or lysosomes, which contain various degradation enzymes^5^. Importantly, LC3-II is also degraded during this step^8^. The last step is the breakdown and recycling of the degraded components in the form of amino acids, lipids, and nucleotides^5^. Altogether, autophagy is important for cell fate and, thus, improper regulation of autophagy is reportedly related to many diseases including cancer, diabetes, and Alzheimer.

Various proteins involved in the autophagic process need zinc for proper functioning^9,10^. Zinc is widely used in eukaryotic cells for DNA-binding of transcription factors, as a structural component, and for the formation of an active site in various enzymes^9,10^. Zinc also play crucial roles for skin homeostasis^11–16^. Growing evidence supports a role of zinc as a positive regulator of autophagy^17,18^. Zinc chelation by the cell-permeable zinc chelator, N,N,N′,N′-Tetrakis(2-pyridylmethyl)ethylenediamine (TPEN), or chelex-100, downregulates autophagy in MCF-cells, astrocytes, and hepatoma cells^18^. TPEN was also found to disrupt lysosomal integrity in macrophages^19^, thereby inhibiting autophagosome formation. The role of zinc in autophagy has just begun to be characterized in detail. Recent studies, based on direct inhibition of phosphatase activity during oxidative stress, revealed the involvement of extracellular-signal-regulated kinases (ERK1/2) in zinc-dependent autophagy^20^. Since zinc homeostasis is regulated by the harmonized actions of zinc transporting proteins and metal scavenger metallothioneins, these factors may be relevant to regulating autophagy for clinical application. However, the impact of zinc regulators on autophagy has just begun to be elucidated.

In this article, based on our previous demonstration that Zrt- and Irt-like protein 13 (ZIP13) is a major zinc transporter expressed in the dermis^21–23^, we investigated the role of ZIP13 in fibrosarcoma. Our genetically modified cell model and mice revealed that modulation of ZIP13-dependent zinc homeostasis could be a future therapeutic strategy to treat fibrosarcoma.

## Results

### *ZIP13* gene expression is downregulated in fibrosarcoma

**Figure 1:**
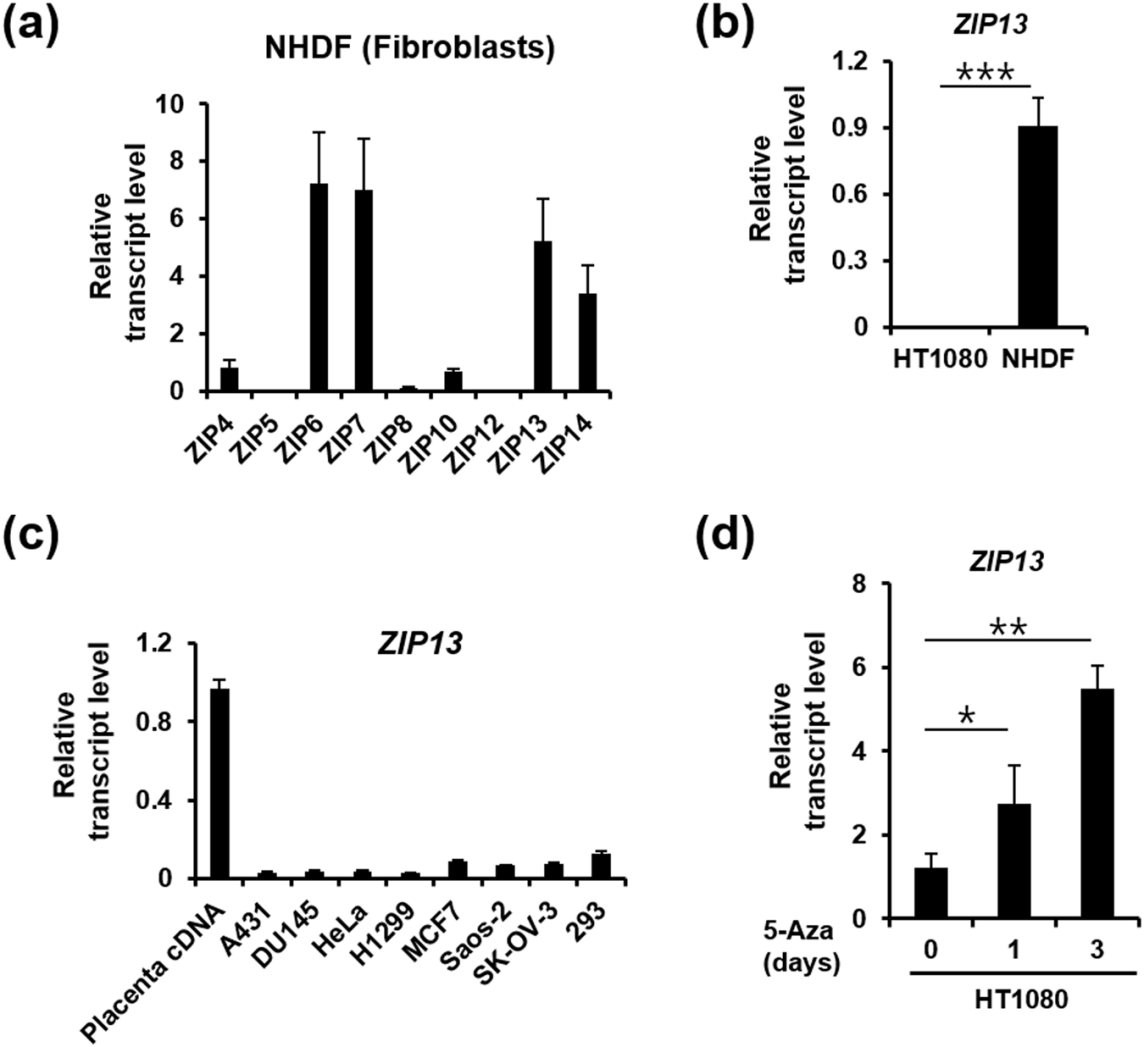
ZIP13 is downregulated in fibrosarcoma. (**a**) Real-time PCR analysis reveals that *ZIP13* is one of the predominant zinc transporter transcripts in normal human fibroblasts. The data represent three independent experiments. (**b**) Real-time PCR analysis reveals that *ZIP13* is downregulated in the HT1080 fibrosarcoma cell line. The data are presented as mean ± SD of three independent experiments (***P < 0.005). (**c**) Real-time PCR analysis reveals that *ZIP13* is downregulated in different cancer cell lines compared to placental cDNA. The data represent three independent experiments. (**d**) Real-time PCR analysis reveals that 5 μM 5-aza-2′-deoxycytidine (5-Aza) treatment increases *ZIP13* expression in the HT1080 fibrosarcoma cell line. The data are presented as mean ± SD of three independent experiments (*P < 0.05, **P < 0.01).

ZIP13 plays a critical role in fibroblasts, and its dysfunction leads to severe dermal disorders^21–23^. Despite the existence, in normal human dermal fibroblasts, of RNA transcripts for other zinc transporters, such as ZIP4, ZIP6, ZIP7, ZIP10, and ZIP14 (Fig. 1a), in normal human dermal fibroblasts, the fact that ZIP13 depletion leads to severe defects in dermis implies the importance of this transporter in fibroblasts^22,24^. To investigate the role of ZIP13 in neoplastic tissues, we compared *ZIP13* expression in normal human dermal fibroblast and in the fibrosarcoma cell line, HT1080. Interestingly, *ZIP13* expression is very low in HT1080 cells (Fig. 1b) and this is also true for other cancer cell lines (Fig. 1c). Based on the fact that DNA methylation is commonly observed in cancer cells^25^, we treated the HT1080 cells with 5-aza-2′-deoxycytidine (5-Aza), a cytidine analog preventing DNA methylation. The 5-Aza treatment increased the expressions of *ZIP13* in the HT1080 cells (Fig. 1d), implying that hypermethylation is involved in *ZIP13* gene repression in these cells and suggesting its possible relationship with fibrosarcoma progression.

### The ZIP13^−/−^ cell line reveals autophagy impairment

**Figure 2:**
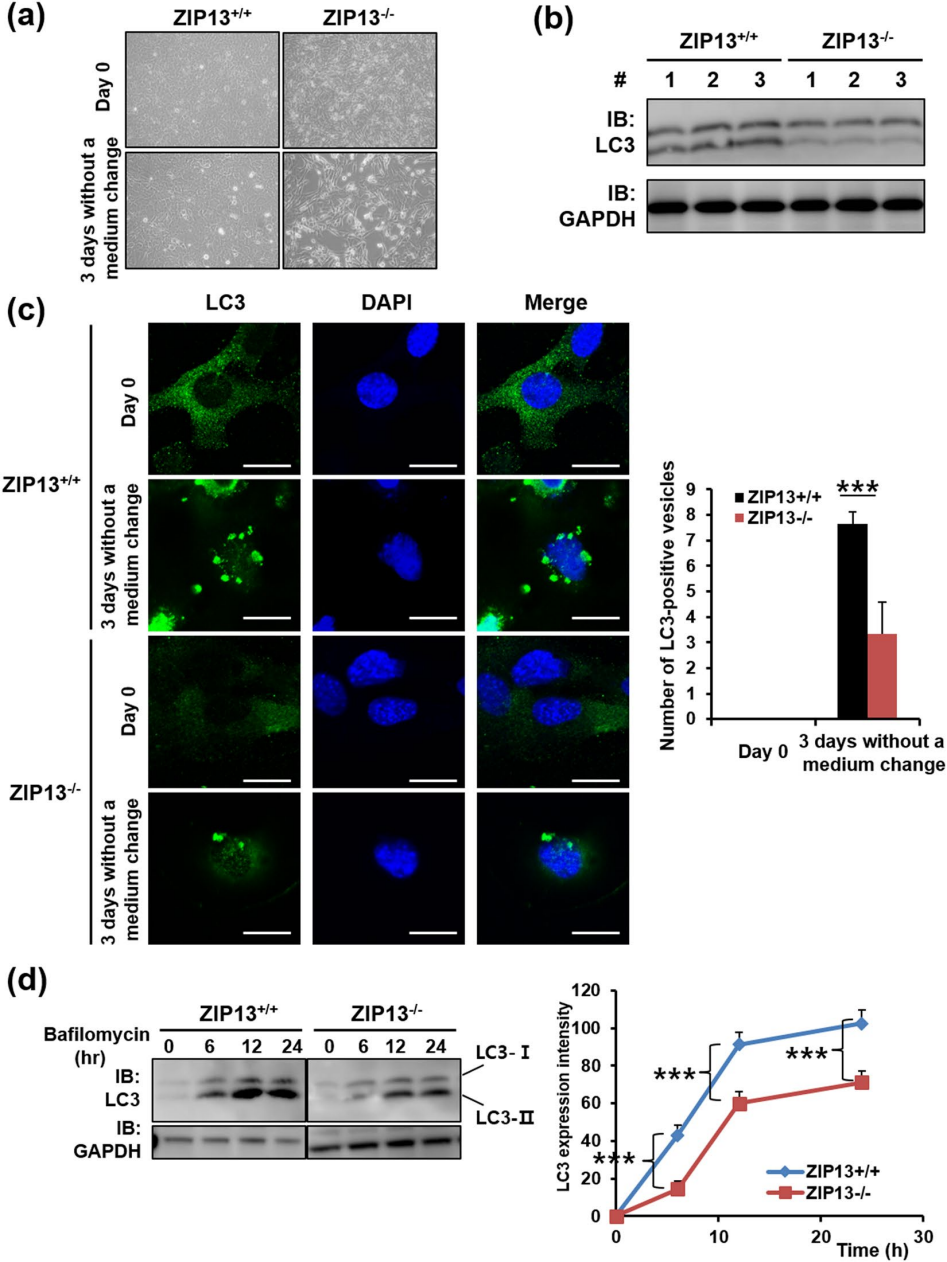
ZIP13^−/−^ cell lines reveal autophagy impairment. (**a**) Microscopic analysis reveals that after 3 days in culture without medium change, floating and spherical cells appear in the ZIP13^−/−^ cultures. (**b**) Western blot analysis reveals the downregulation of LC3 proteins in the ZIP13^−/−^ cell line. Uncropped blots are shown in Fig. S1b. (**c**) Confocal microscopic analysis reveals the decrease in LC3-positive vacuoles in the ZIP13^−/−^ cell line after 3-day culturing without medium change (n = 5, ***P < 0.005). Scale bars = 25 μm. (**d**) Western blot analysis shows that bafilomycin treatment does not restore the accumulation of LC3 proteins in the ZIP13^−/−^ cell line. The relative intensity of total LC3s was measured by the ImageJ software (http://rsbweb.nih.gov/ij/download.html). The data represent three independent experiments (***P < 0.005). Uncropped blots are shown in Fig. S2.

To elucidate the effects of ZIP13 repression in fibrosarcoma, two cell line models were generated. Wild-type and ZIP13-depleted dermal fibroblasts were isolated from mice^24^ and transfected with plasmids encoding simian vacuolating virus 40^26^. The transfected cell lines were called ZIP13^+/+^ and ZIP13^−/−^, respectively. The quantitative RT-PCR analysis revealed that *Zip13* expression was successfully knocked out in the ZIP13^−/−^ cell line (Fig. S1a). After 3 days culturing without medium change, floating and spherical cells selectively appeared in the ZIP13^−/−^ cultures (Fig. 2a). Notably, these conditions are expected to induce autophagy as a result of nutrient depletion. However, western blot analysis using the anti-LC3 antibody revealed that the expression of LC3, an autophagic marker, was significantly reduced in the ZIP13^−/−^ cell line (Fig. 2b). Confocal microscopy showed that, after 3 days in culture without medium change, autophagic puncta were induced in both cell lines, but their number was remarkably lower in the ZIP13^−/−^ cell line, compared to the ZIP13^+/+^ cell line (Fig. 2c). To precisely compare LC3 induction and LC3-I conversion to LC3-II in both cells, we treated cells with bafilomycin A, a well-known lysosomal protease inhibitor that inhibits late autophagic events and autophagosome-lysosome fusion. If Bafilomycin A treatment increased the LC3-II level after autophagy induction, autophagic flux would be enhanced. If not, blockade of autophagosome-lysosome fusion would be expected^6^. The treatment resulted in the accumulation of LC3-II in both cell lines, but the LC3-II protein levels were consistently lower in the ZIP13^−/−^ cell line (Fig. 2d). Bafilomycin A treatment caused a greater increase in the LC3-II level in the autophagy-induced ZIP13^−/−^ cell line than in the non-induced ZIP13^−/−^ cell line (Fig. S3). These data imply a decline in autophagic flux in the ZIP13^−/−^ cell line compared to the ZIP13^+/+^ cell line and that LC3 expression might be transcriptionally decreased in the ZIP13^−/−^ cell line. Moreover, we found that the expression of proteins such as ATG14L and WIPI2 that are involved in phagosome formation for membrane isolation, which is an early stage in autophagy process, were comparable between the ZIP13^−/−^ and the ZIP13^+/+^ cell lines (Figure S4a,b). This supports the hypothesis that the decline in autophagic flux in the ZIP13^−/−^ cell line is strongly related to reduced LC3 expression.

### The ZIP13^−/−^ cells have a defect in LC3 expression

**Figure 3:**
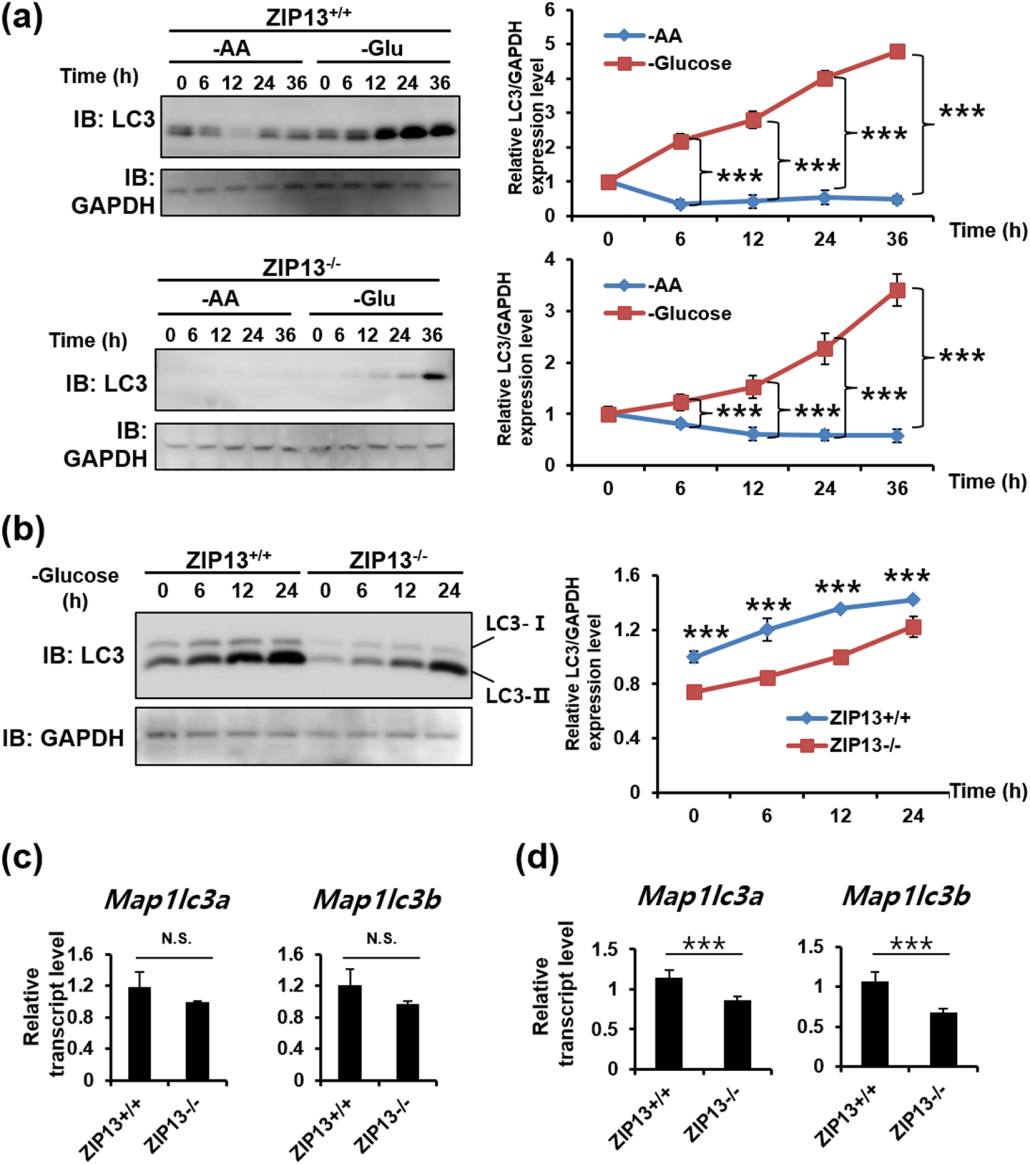
ZIP13^−/−^ cell lines have a defect in autophagy induction. (**a**) Western blot analysis reveals that glucose depletion induces LC3 expression. –AA, amino acid depletion; -Glu, glucose depletion. Uncropped blots are shown in Fig. S5. (**b**) Western blot analysis reveals that glucose depletion significantly induces LC3 expression in the ZIP13^+/+^ cell line compared to the ZIP13^−/−^ cell line. Uncropped blots are shown in Fig. S6. (**c**,**d**) Real-time PCR analysis reveals that the expression levels of *Map1lc3a* and *Map1lc3b* are comparable in both cell lines under normal condition (**c**). However, glucose depletion-induced *Map1lc3a* and *Map1lc3b* upregulation is significantly stronger in the ZIP13^+/+^ compared to the ZIP13^−/−^ cell line. The relative intensity of total LC3s was measured by ImageJ software (http://rsbweb.nih.gov/ij/download.html). All data in graphs represent three independent experiments.

LC3 induction and LC3-I conversion to LC3-II are triggered by nutrient depletion^8^. To accurately monitor LC3 induction and LC3-I conversion to LC3-II in the ZIP13^+/+^ and ZIP13^−/−^ cell lines, we compared two methods for autophagy induction. Amino acid and glucose depletions are well-known nutrient-based autophagy induction methods. Fresh medium was added 24 h before autophagy induction. The results revealed that amino acid depletion did not affect LC3 expression (Fig. 3a). However, glucose depletion induced LC3 expression in both cells, and more significantly in the ZIP13^+/+^ cell line compared to the ZIP13^−/−^ cell line (Fig. 3b). Glucose depletion increased LC3-I conversion to LC3-II in both cells in a time-dependent manner (Fig. 3b). However, the LC3 protein levels in the ZIP13^−/−^ cell line were consistently low, as compared to the ZIP13^+/+^ cell line (Fig. 3a,b). We next analyzed the induction of *LC3* transcripts before and after glucose depletion. Under basal conditions, the levels of both known *LC3* transcript isoforms, *Map1lc3a* and *Map1lc3b*, were slightly lower in the ZIP13^−/−^ cell line, albeit the differences were not statistically significant (Fig. 3c). However, upon glucose depletion, a substantial and statistically significant reduction in both transcripts was observed in the ZIP13^−/−^ cell line compared to the ZIP13^+/+^ cell line (Fig. 3d). Therefore, *LC3* gene repression by an undefined mechanism may occur in the ZIP13^−/−^ cell line, contributing to reduced autophagosome formation, compared to the ZIP13^+/+^ cells.

### DNA methyltransferase activity is changed in the ZIP13^−/−^ cell line

**Figure 4:**
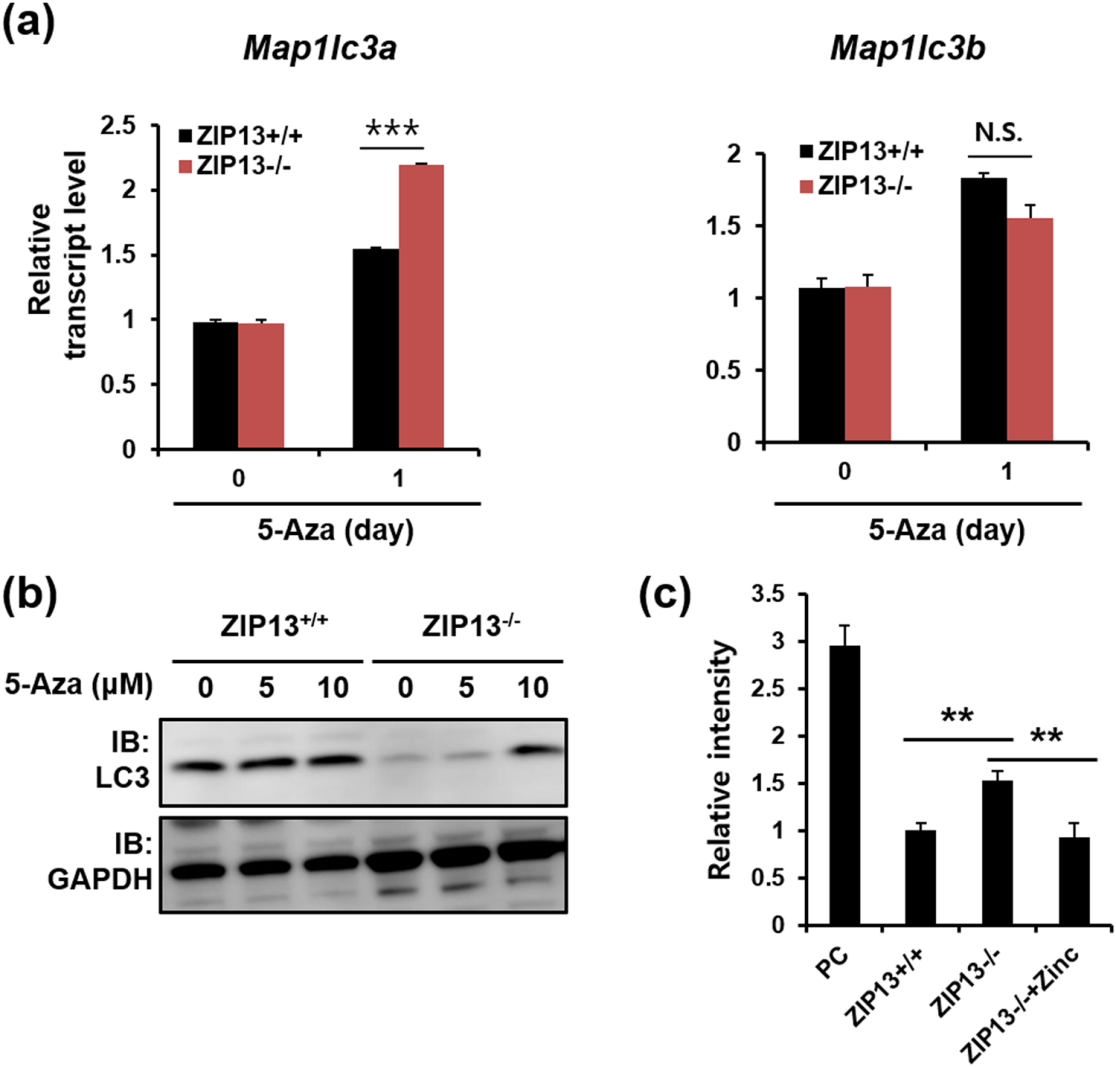
5-Aza treatment recovers the ZIP13 expression. (**a**) Real-time PCR analysis reveals that 5-Aza treatment induces *Map1lc3a* expression more significantly in the ZIP13^−/−^ cell line compared to the ZIP13^+/+^ cell line. The data are presented as mean ± SD of three independent experiments (***P < 0.005). (**b**) Western blot analysis reveals that 5-aza-2′-deoxycytidine (5-Aza) treatment for 24 h increases ZIP13 expression in the ZIP13^−/−^ cell line. Uncropped blots are shown in Fig. S7. (**c**) The DNA methyltransferase (DNMT) assay reveals that the DNMT activity is higher in the ZIP13^−/−^, compared to the ZIP13^+/+^ cell line. The data are presented as mean ± SD of three independent experiments (**P < 0.01). The Y-axis shows the relative intensity of the OD 450 nm value with respect to the ZIP13 + / + signal, which was set at 1.

As DNA methylation is a well-known gene repression mechanism in cancer cells, we investigated whether the 5-Aza treatment restored the reduced *LC3* gene expression in the ZIP13^−/−^ cell line. The 5-Aza treatment inhibits DNA methylation, allowing for the re-expression of hypermethylation-repressed genes^25^. In both ZIP13^+/+^ and ZIP13^−/−^ cell lines, *LC3* expression is increased after the 5-Aza treatment. Notably, the increase in *Map1lc3a* expression is more evident in the ZIP13^−/−^ cell line than in the ZIP13^+/+^ cell line (Fig. 4a). Western blot analysis with anti-LC3 antibody revealed that the 5-Aza treatment increased the LC3 protein level in the ZIP13^−/−^ cell line (Fig. 4b). Next, DNA methylation regulatory proteins—methyltransferases (DNMTs)—were comparatively analyzed in the ZIP13^+/+^ and ZIP13^−/−^ cell lines. Although the protein and mRNA expressions of methyltransferases (DNMTs) were comparable between the ZIP13^+/+^ and ZIP13^−/−^ cell lines (Figs S8, S9), DNMT activity was found to be significantly elevated in the ZIP13^−/−^ cell line (Fig. 4c). Moreover, as previously reported for other ZIP13-depleted or -mutated cells, the intracellular zinc level was significantly decreased in the ZIP13^−/−^ cell line (Fig. S10), and zinc addition reverted the DNMT activity level in the ZIP13^−/−^ cell line to that in the ZIP13^+/+^ cell line (Fig. 4c). These results indicate that the reduced intracellular zinc level in the ZIP13^−/−^ cell line disrupts DNMT activity for DNA methylation.

### TPEN treatment induces fibrosarcoma death with low damages on the normal cells

**Figure 5:**
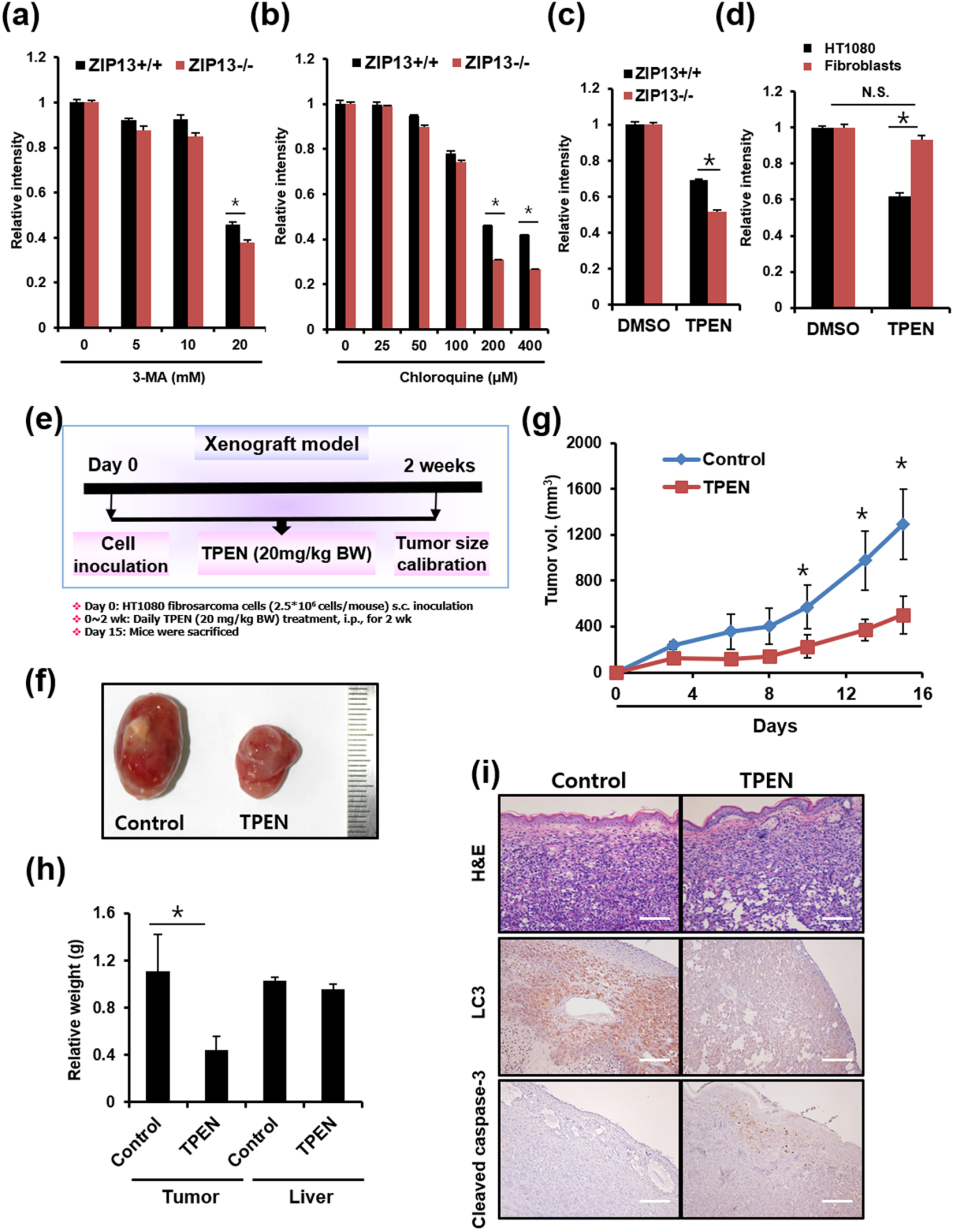
TPEN treatment induces fibrosarcoma death in a mice model. (**a**–**d**) Crystal violet assay reveals that 3-MA, chloroquine, and TPEN treatment lead to more severe cytotoxicity in the ZIP13^−/−^ cell line and the HT1080 fibrosarcoma cell line, compared to the ZIP13^+/+^ cell line, as well as normal human dermal fibroblast. The data are presented as mean ± SD of three independent experiments (*P < 0.05). (**e**) Flowchart of HT1080 fibrosarcoma xenograft model. (**f**) Tumor image representative of 5 independent experiments. (**g**,**h**) Tumor volume and weight were significantly decreased by TPEN treatment compared to the control (*P < 0.05). (**i**) IHC analysis showed that TPEN treatment decreased LC3 and increased the level of cleaved caspase 3, compared to the control in tumor tissue. Scale bars = 500 μm.

Based on our evidence of defective autophagy in the ZIP13^−/−^ cells, we employed two different well-known autophagy inhibitors, 3-methyladenine (3-MA) and chloroquine^8^, to induce selective death of fibrosarcoma. 3-MA blocks autophagosome formation by inhibiting the phosphatidylinositol 3-kinases (PI3K). Chloroquine accumulates inside the acidic lumen of the intracellular compartments including endosomes and lysosomes, leading to pH elevation, thereby inhibiting the fusion of autophagosome with lysosome for protein degradation. Both ZIP13^+/+^ and ZIP13^−/−^ cell lines were treated with 3-MA or chloroquine, and cytotoxicity was monitored (Fig. 5a,b). As expected, autophagy inhibitors exerted higher cytotoxicity in the ZIP13^−/−^ than the ZIP13^+/+^ cell line (Fig. 5a,b). Thus, we reasoned that a possible therapeutic approach could consist in the pharmacological control of both autophagy and zinc homeostasis. To investigate this, we applied TPEN, one of the most well-known and strongest zinc-specific chelators. Recent advances indicate that zinc depletion by TPEN blocks autophagy^17,18^. The TPEN treatment exhibited a higher cytotoxicity in the ZIP13^−/−^ cell line compared to the ZIP13^+/+^ cell line (Fig. 5c). In addition, we found that TPEN exerted strong cytotoxicity in the HT1080 cell line at concentrations that did not significantly affect the viability of normal human dermal fibroblast (Fig. 5d), suggesting the possible use of TPEN in fibrosarcoma therapy.

Next, TPEN effects were tested in a mice fibrosarcoma xenograft model (Fig. 5e). Specifically, we investigated the effects of TPEN on the growth of HT1080 fibrosarcoma cells transplanted into male Balb/c nude mice. The tumor growth volume was significantly inhibited in mice treated with TPEN (20 mg/kg BW, i.p.), compared to vehicle-treated control mice (Fig. 5f,g). TPEN also significantly decreased the average tumor weight without changes in liver weight. After 2 weeks, we also examined the expressions of LC3 and cleaved caspase 3 by IHC analysis in tumor tissue. The TPEN treatment group exhibited decreased LC3 expression and increased cleaved caspase 3 expression (Fig. 5i). These results indicated that TPEN treatment not only decreased autophagy but also induced apoptosis in fibrosarcoma.

## Discussion

**Figure 6:**
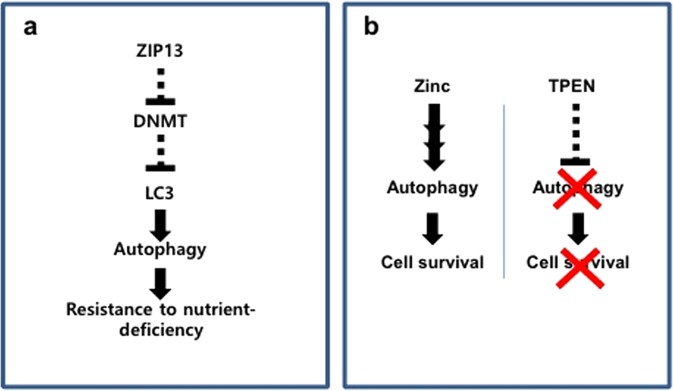
Models for ZIP13 and zinc involvements in fibrosarcoma survival. (**a**) ZIP13 causes resistance to nutrient-deficiency in fibrosarcoma. (**b**) Zinc supports autophagy. When TPEN is applied, proper autophagy is blocked, resulting in reduced cell survival.

In this study, we found that ZIP13 is abundantly expressed in normal fibroblasts, but down-regulated in various cancer cells including a fibrosarcoma. The ZIP13^−/−^ cell line showed reduced autophagy induction after glucose starvation, compared to the ZIP13^+/+^ cells. Moreover, the ZIP13^−/−^ cells exhibited defective LC3 expression due to altered DNMT activity, following to hypersensitivity to nutrient deficiency (Fig. 6a). TPEN, a well-known zinc chelator and putative autophagy inhibitor, proved to be an effective and safe anti-cancer drug by selectively killing fibrosarcoma through both inhibitions of autophagy and chelation of zinc (Fig. 6b).

Autophagy is a eukaryotic conserved systemic adaptation process allowing for cell life prolongation under conditions of nutrient deficiency^27^. Recent advances have demonstrated the multifaceted role of autophagy in cell maintenance, development, differentiation, and aging^7,28–30^, and its relations with diverse human diseases including cancer, Alzheimer’s disease, and pathogenic infection. The LC3 protein is encoded by two genes, named *Map1lc3a* and *Map1lc3b*^31^. The treatment with 5-Aza restored the mRNA expression of *Map1lc3a*, but not *Map1lc3b*, in the ZIP13^−/−^ cell line. These data imply that indirect involvement of methylation might be associated with the protein level of LC3 such as affecting other molecules that regulate the protein stability or degradation. The two genes are located on distinct chromosomes, chromosome 20 and 16, exhibit a cell type-specific expression pattern, and their post-translational modifications are reportedly distinct^31,32^. Thus, the expression of the two *Map1lc3* isoforms is subject to independent and complex regulation by epigenetic and enzymatic events, in response to autophagy induction. Other mechanisms, possibly responsible for *Map1lc3b* suppression in the ZIP13^−/−^ cell line, must still be elucidated. In addition, we found that p62, which is an autophagosome cargo protein, is continuously downregulated in the ZIP13^−/−^ cell line similar to the LC3 protein (Fig. S11), but not restored by the 5-Aza treatment (Figure S12), implying that the loss of ZIP13 leads to changes in the autophagy-related protein expression. ZIP13 expression was found to be downregulated in HT1080 cells, as compared to normal fibroblasts, but was restored by the 5-Aza treatment. Epigenetic gene silencing is a well-known phenomenon in cancers. Indeed, tumor suppressor genes including p16 are often inactivated in various cancers due to methylation of cytosine residues in CpG sequences, which is the most well-known DNA modification associated with epigenetic gene silencing^25^. Recent studies have demonstrated that ZIP8 expression is suppressed in cadmium-resistant metallothionein-null (A7) cells by the hypermethylation of the CpG island of the *slc39a8* gene^33^, and long-term exposure to cadmium results in enhanced DNA methylation by the elevation of DNMTs^34^. Based on the hypothesis that zinc imbalance disturbs DNA methylation, zinc deficiency or excess might lead to similar phenotypes to those characterizing the human genetic disorders, ICF (immunodeficiency, centromeric instability and facial anomalies) syndrome and Rett syndrome, associated to mutations in the *DNMT3B* and *MECP2* gene, respectively^35^. DNMT is composed of two domains, i.e., the N-terminal domain, which suppresses de novo methylation, and the C-terminal domain, which is the catalytic domain^35^. The N-terminal domain contains a Cys-rich region that can bind zinc. Although the precise function of the Cys-rich region is unknown, zinc binding to this region has been suggested to inhibit DNA methylation by either directly interfering with DNMT activity or by preventing proper enzyme access to DNA, thus explaining the observed zinc-induced inhibition of *in vitro* DNMT activity^36^. In accordance with ZIP8 and ZIP13 suppression, many human transporters that mediate the influx of diverse substrates including monocarboxylate, organic anions, glutamate, serotonin, and folate are also repressed by DNA methylation^37–40^. Treatment with 5-Aza could counteract these effects, restoring substrate influx and chemical balance. Therefore, 5-Aza could be employed to regulate altered transport in various diseases and may represent a new therapeutic strategy.

The potential application of TPEN as an anti-cancer drug has been explored by several groups^41,42^. In cellular models of breast cancer, treatment with a cell-permeable zinc chelator, TPEN led to apoptosis due to elevation of caspase activity, caused by chromatin condensation and nuclear fragmentation^41^. This phenomenon was also observed in prostate cancer cell models. TPEN treatment activates caspase 3/7, followed by Poly ADP ribose polymerase (PARP) cleavage and DNA fragmentation^42^. Although the caspase species activated by zinc chelation in breast and prostate cancer cells are different, zinc chelation can successfully activate diverse caspase family members^43^. Our previous report showed that TPEN activates caspase 3, 8, 9 and 12 in B cells^44^. Caspases are cysteine proteases possessing many zinc-accessible residues, including multiple amino acids with affinity for zinc, such as cysteines and histidines. Zinc directly binds to these residues either within the active catalytic sites or at distant allosteric sites of caspases^45,46^. Therefore, clinical trials with TPEN could pave the way to novel therapeutic approaches for cancer.

In the present study, we identified a role for ZIP13 in fibrosarcoma. ZIP13 expression is epigenetically suppressed in fibrosarcoma, causing defective induction of autophagy and decreased resistance to nutrient deficiency. Finally, we propose TPEN as a possible anti-fibrosarcoma drug. TPEN exerts lower toxicity in normal cells and tissues compared to cancer cells and tumors. The applicability of TPEN in fibrosarcoma therapy and, more in general, the suitability of therapeutic approaches based on the pharmacological regulation of zinc balance within cells and tissues, deserve further investigation.

## Methods

### Cell culture and materials

Mice dermal fibroblasts were isolated as previously described^22,24^, co-transfected with pSV40 expressing plasmids and pZeocin (pSV40: pZeocin = 10:1), and screened by Zeocin treatment (100–500 μg/ml) as previously described^26^. The HT1080 cell line was purchased from the Korean Cell Line Bank (Seoul, Korea), and maintained in DMEM medium (Gibco) with 10% FBS and antibiotics, at 37 °C. Normal human fibroblasts were purchased from Lonza (Basel, Switzerland) and maintained in DMEM medium (Gibco) with 10% FBS and antibiotics, at 37 °C. cDNAs of cancer cell lines were purchased from Takara (Shiga). 5-aza, bafilomycin and TPEN were purchased from Sigma, and dissolved in DMSO.

### Xenograft assay

Animal care and studies were performed in accordance with the Guide for the Care and Use of Laboratory Animals of the National Institutes of Health. This animal care and use protocol was reviewed and approved by the Institutional Animal Care and Use Committee (IACUC) at Yeungnam University College of Medicine (number: YUMC-AEC2017-016). Male Balb/c nude mice (6 weeks old) were purchased from Orient Bio Inc. (Seongnam, Gyeonggi-do, South Korea) and maintained in micro-isolator cages in a pathogen-free facility under a 12 h light-dark cycle at 22–24 °C and 50% humidity. Animals were fed with *ad libitum* diet and water. After a 1-week acclimatization with basal diet, mice were subcutaneously inoculated with exponentially growing HT1080 fibrosarcoma cells (2.5 × 10^6^ cells in 100 μl PBS) on the backbone area. Mice were randomly divided into groups of five animals each. TPEN (20 mg/kg BW) or vehicle was supplied daily by i.p. injection for 2 weeks. The experiment was terminated 2 weeks after tumor cell injection. The tumor volume was calculated by the formula (length × width^2^)/2. Immunohistochemistry to evaluate LC3 and cleaved caspase 3 (Cell Signaling Technology) was performed using excised tumor tissue.

### DNMT assay

DNMT assay was performed as the manufacturer’s instruction (Abcam). Briefly, Nucelar extracts were purified by nuclear extraction kit (Abcam), and incubated with substrate and assay buffer, and then incubated with capture antibody, followed to the addition of detector antibody and enhancer solution. The absorbance was read on a microplate reader at 450 nm with an optional reference wavelength of 655 nm.

### Crystal violet assay

Crystal violet assay was performed as previously described^47^. Cells were treated with TPEN, and further incubated for 24 h. On the assay day, cells were fixed by 4% paraformaldehyde (PBS), and then stained with 500 µL of 0.1% crystal violet.

### Fluorescence microscopy

For fluorescence microscopy, cells were cultured on Lab-Tek chamber slides (Thermo Fisher Scientific) and stained with Anti-LC3B (Cell Signaling Technology) as previously described^48^.

### Quantitative RT-PCR

RNAs were extracted with the RNAeasy Kit (Qiagen) according to the manufacturers’ instructions. cDNA was synthesized as previously reported^49^. The mRNA levels of *ZIP13* (Hs00378317_m1), *Zip13* (Mm01329757_m1), *Map1lc3a* (Mm00782868_sH), and *Map1lc3b* (Mm00458724_m1) were analyzed using the TaqMan Gene Expression Assay following the manufacturer’s instructions (Thermo Fisher Scientific). Briefly, Sample expression levels were normalized to *GAPDH* expression levels according to the 2^−ΔΔCt^ method, where ΔCt = Ct of the target gene – Ct of *GAPDH*, and ΔΔCT = ΔCT of the target sample – ΔCT of the Control sample.

### Western blot analysis

Cells were harvested in LIPA buffer (Sigma) as previously reported^13^. After centrifugation at 15,000 × g for 5 min, the supernatant was boiled for 5 min in SDS-PAGE sample buffer (Wako) and loaded onto 5–20% gradient or 15% gels for 1~2 h. Proteins were transferred to nitrocellulose membranes (Bio-Rad) by using the Trans-Blot Electrophoretic Transfer cell (Bio-Rad). The membrane was washed in 20 mM Tris-HCl buffer with 1% Tween 20 (TBS-T) (Sigma-Aldrich), incubated for 1 h in blocking buffer (5% milk powder (Merck) in TBS-T), and then further incubated overnight with primary antibodies; Anti-LC3 (Cell signaling) and anti-GAPDH (Santa Cruz) antibodies were purchased and diluted in blocking buffer by 1:3000. The horseradish peroxidase (HRP)-labeled secondary antibody (Dako) was diluted by 1:5000 in TBS-T for secondary antibody staining for 1 h at RT. The membrane was then washed in TBS-T and the signals were developed using the Lumi-Light reagent (Roche) for 5 min. The signal was detected on the LAS-3000 imaging system (Fuji).

### Statistics

The two-tailed Student’s t-test was used to analyze the difference between two groups.

## Supplementary information

Supplemental Info

## Data Availability

Please contact the corresponding author for all data requests.
