# Supplementary material for: Loss of the dermis zinc transporter ZIP13 promotes the mildness of fibrosarcoma by inhibiting autophagy
Source: Sci Rep. 2019 Oct 21;9:15042. doi: 10.1038/s41598-019-51438-9 (PMC6803768; doi:10.1038/s41598-019-51438-9)
Supplement: Supplementary file 1 — Supplemental Info [file 41598_2019_51438_MOESM1_ESM.pdf]

Loss of the dermis zinc transporter ZIP13 promotes the mildness of fibrosarcoma by inhibiting autophagy

Mi-Gi Lee, Min-Ah Choi, Sehyun Chae, Mi-Ae Kang, Hantae Jo, Jin-myoung Baek, Kyu-Ree In, Hyein Park, Hyojin Heo, Dongmin Jang, Sofia Brito, Sung Tae Kim, Dae-Ok Kim, Jong-Soo Lee, Jae-Ryong Kim and Bum-Ho Bin

a

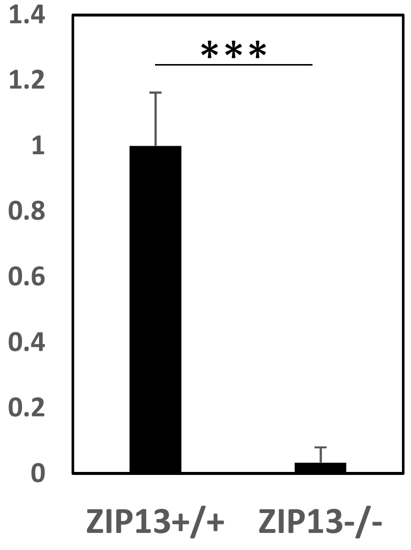

b

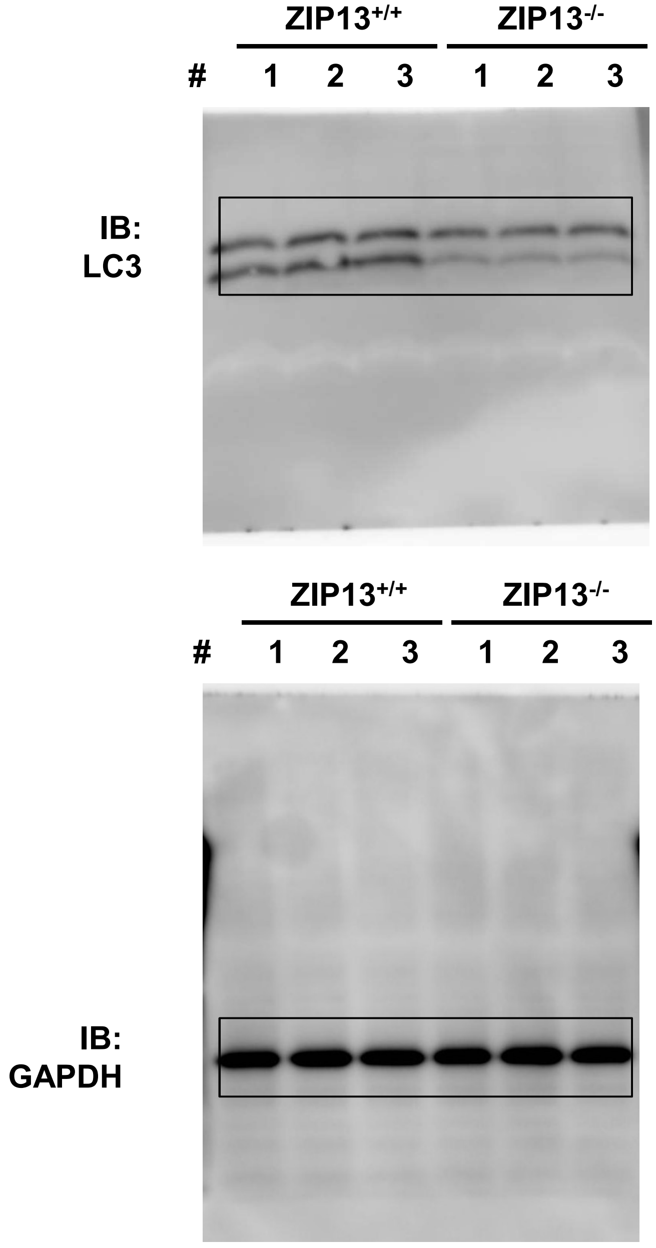

Figure S1. (a) Real-time PCR analysis revealed that ZIP13 expression was knocked out in the $ZIP13^{-/-}$ cell line. The data represent the results of three independent experiments (*, $P < 0.005$). (b) Western blot analysis revealed downregulation of LC3 proteins in the $ZIP13^{-/-}$ cell line.**

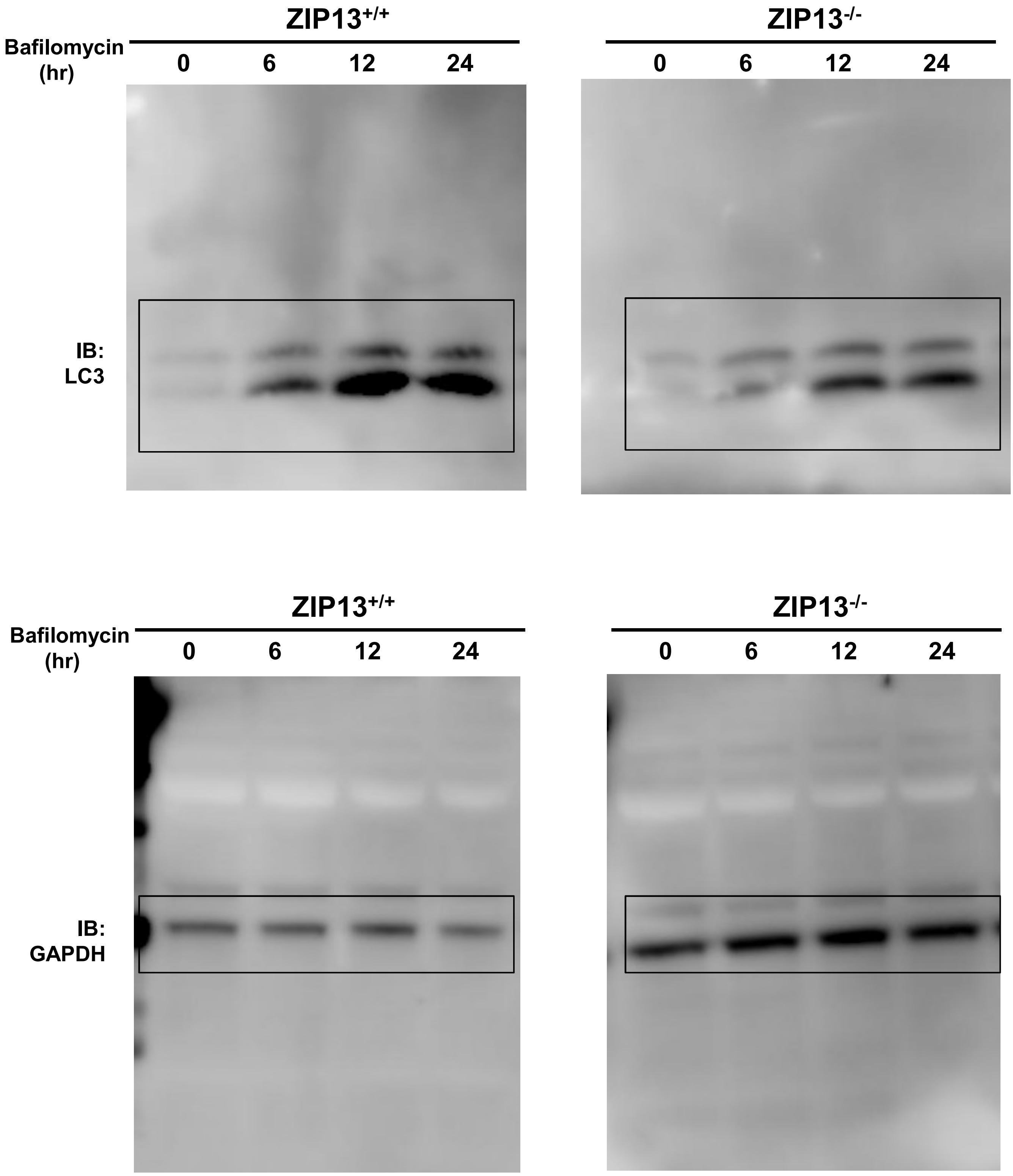

Figure S2. Western blot analysis showed that bafilomycin treatment does not restore the accumulation of LC3 proteins in the ZIP13$^{-/-}$ cell line.

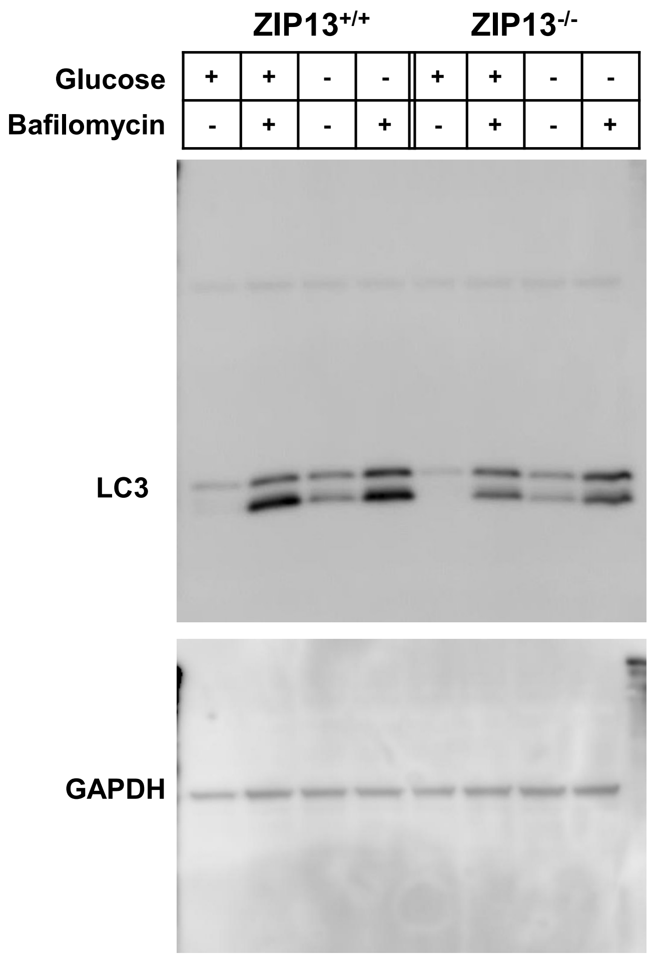

Figure S3. Western blot analysis revealed that bafilomycin A treatment reversed the LC3 expression level in the ZIP13$^{-/-}$ cell line to a lesser extent than in the ZIP13$^{+/+}$ cell line. Cells were incubated with 1 µM bafilomycin for 24 hr with or without glucose, and western blot analysis was then performed.

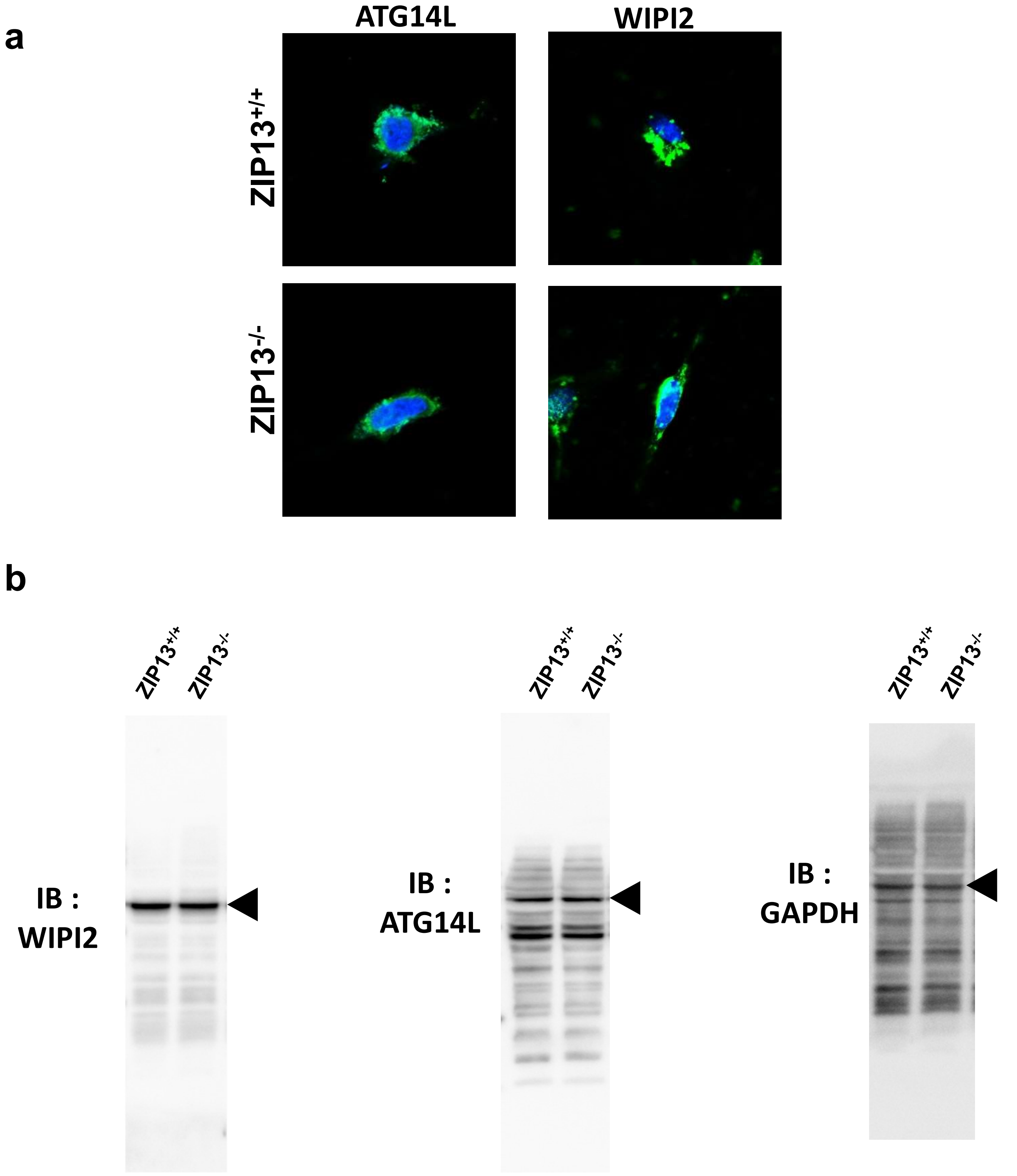

Figure S4. (a) Confocal microscopy analysis revealed that WIPI2 and ATG14L expression was comparable between the ZIP13$^{+/+}$ and ZIP13$^{-/-}$ cell lines. (b) Western blot analysis revealed that WIPI2 and ATG14L expression was comparable between the ZIP13$^{+/+}$ and ZIP13$^{-/-}$ cell lines.

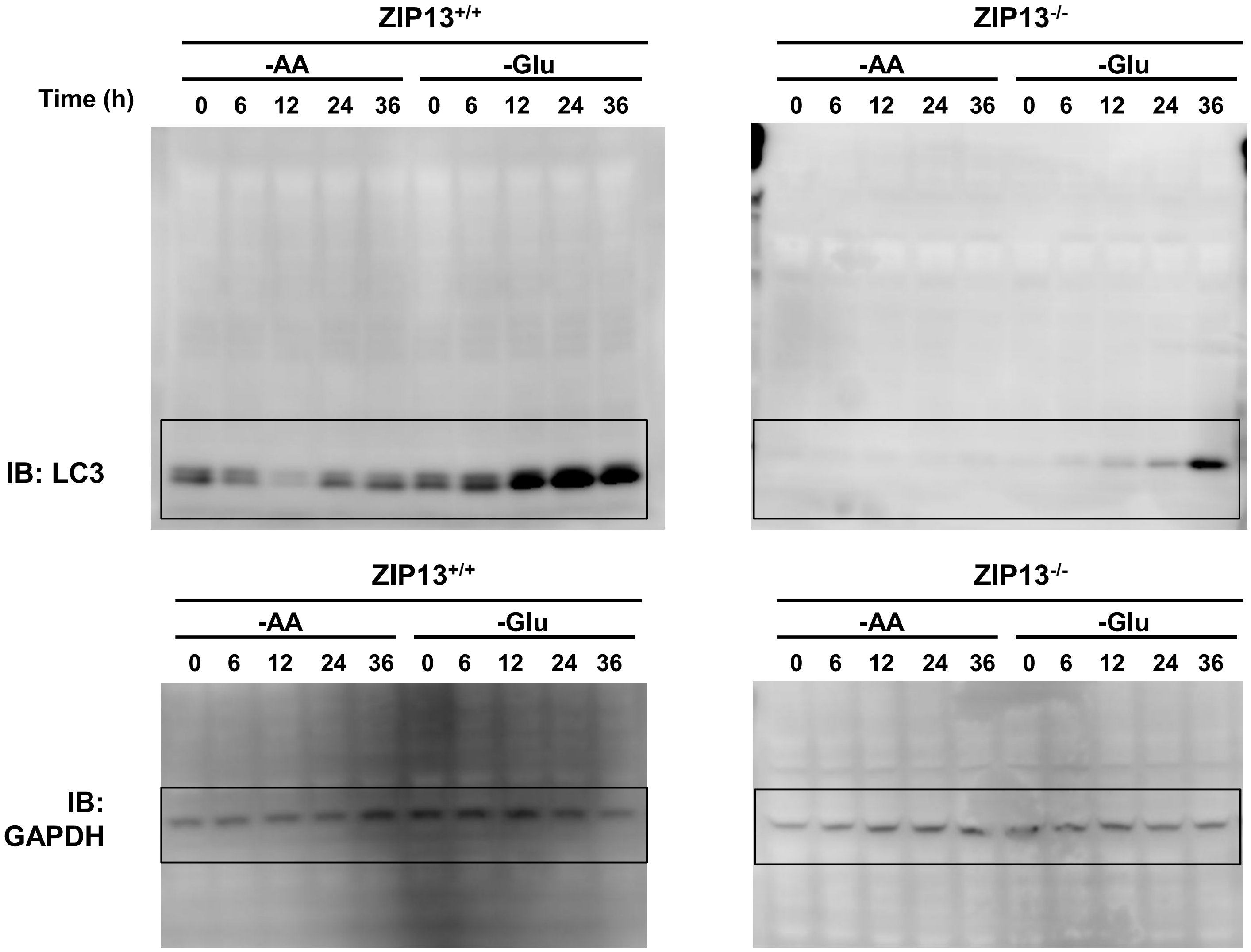

Figure S5. Western blot analysis revealed that glucose depletion induces LC3 expression. –AA, amino acid depletion; -Glu, glucose depletion.

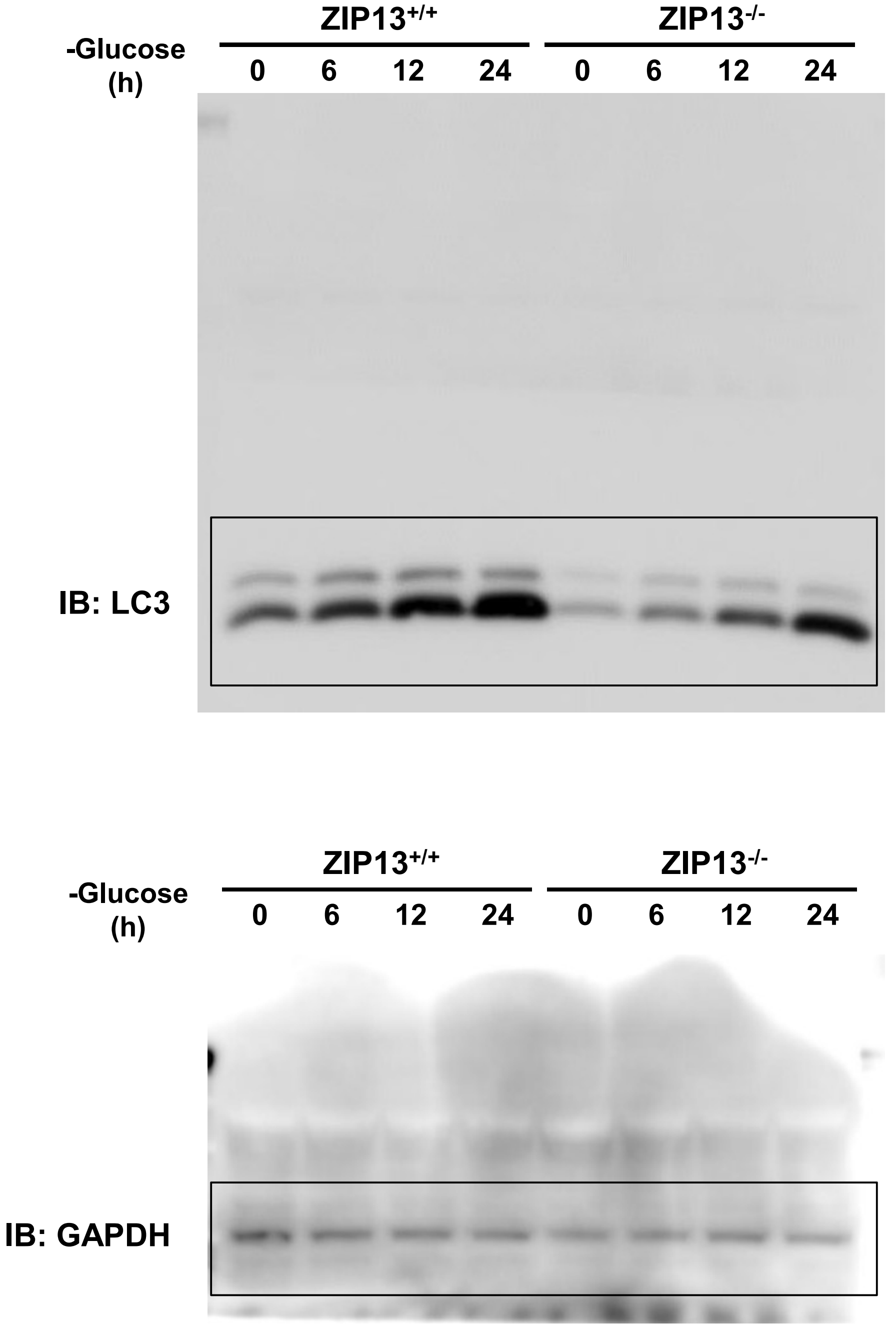

Figure S6. Western blot analysis revealed that glucose depletion significantly induces LC3 expression in the ZIP13$^{+/+}$ cell line compared to the ZIP13$^{-/-}$ cell line.

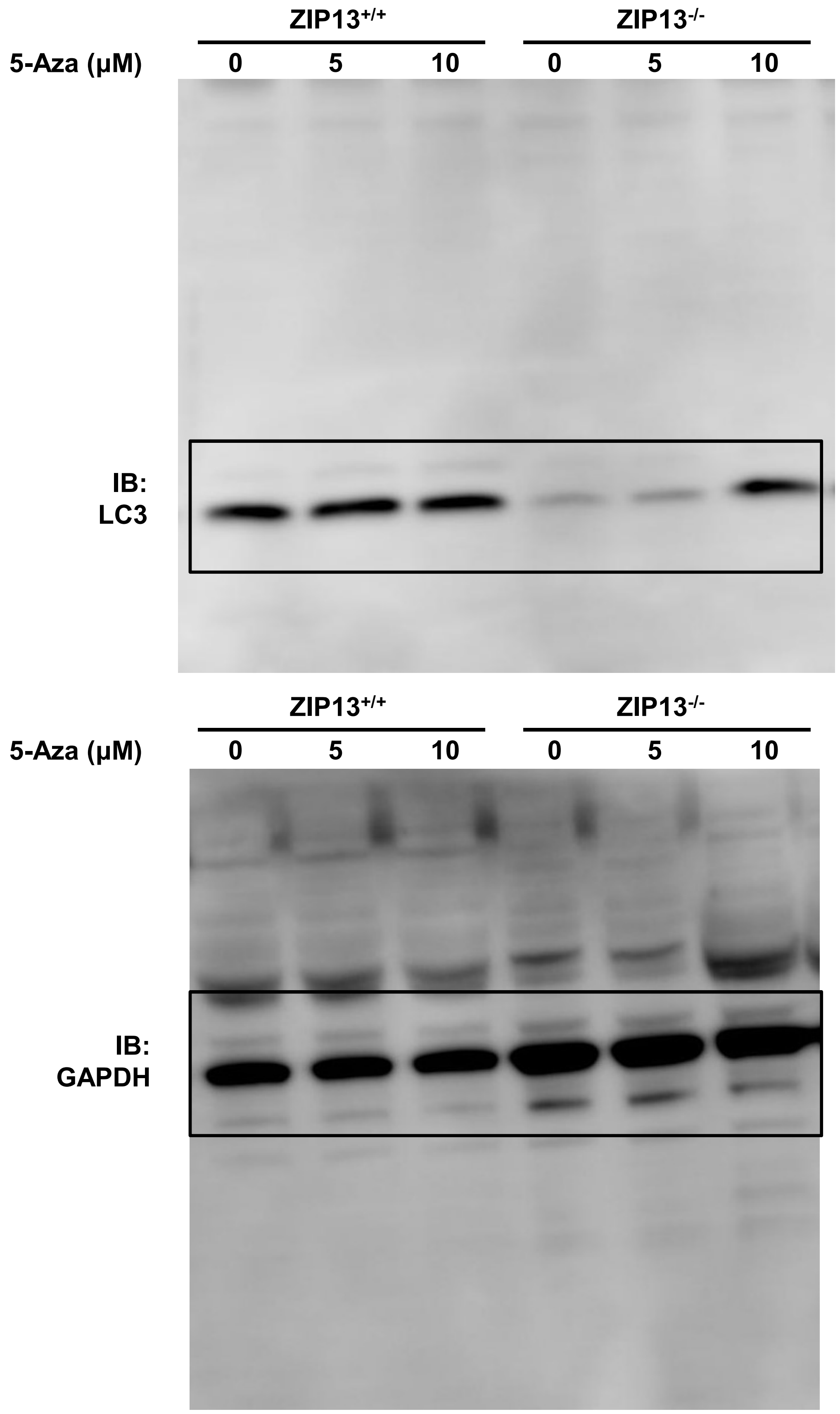

Figure S7. Western blot analysis revealed that 5-aza-2'-deoxycytidine (5-Aza) treatment for 24 h increases ZIP13 expression in the ZIP13$^{-/-}$ cell line.

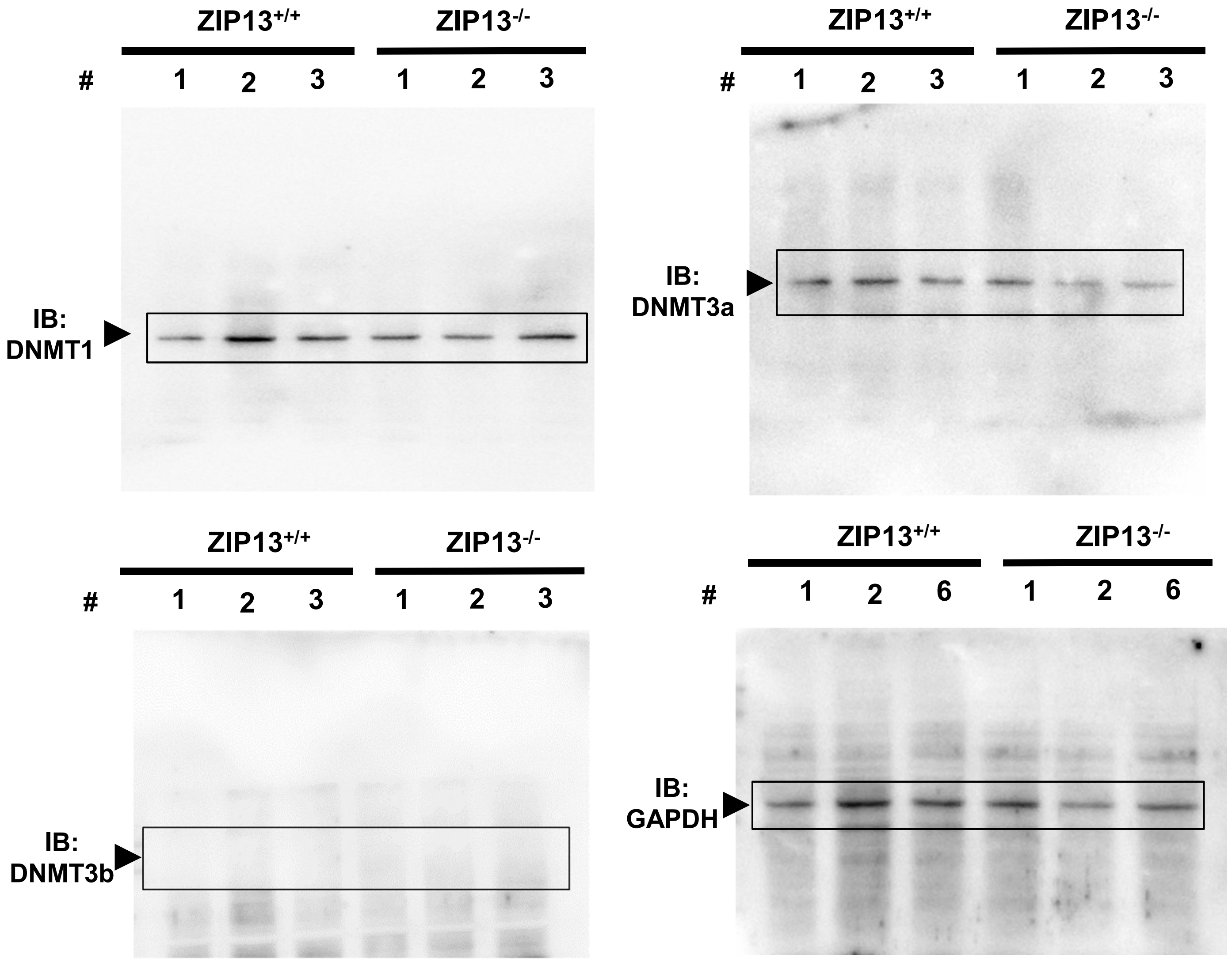

Figure S8. Western blot analysis revealed that the expression levels of DNMTs were comparable between the ZIP13$^{+/+}$ and ZIP13$^{-/-}$ cell lines.

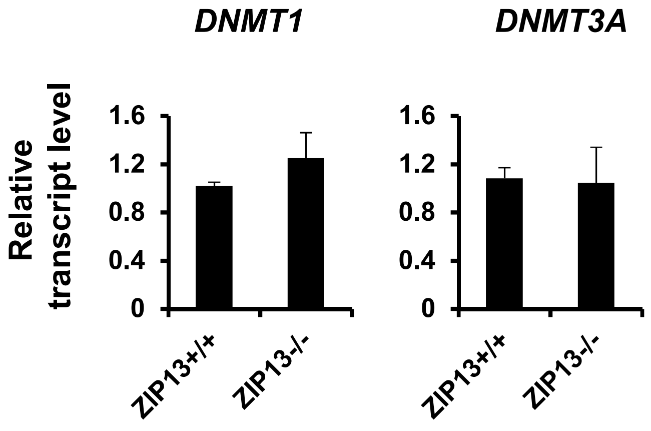

Figure S9. Real-time PCR analysis revealed that DNMT expression was comparable between the ZIP13$^{+/+}$ and ZIP13$^{-/-}$ cell lines. The data represent the results of three independent experiments (*, P < 0.005).**

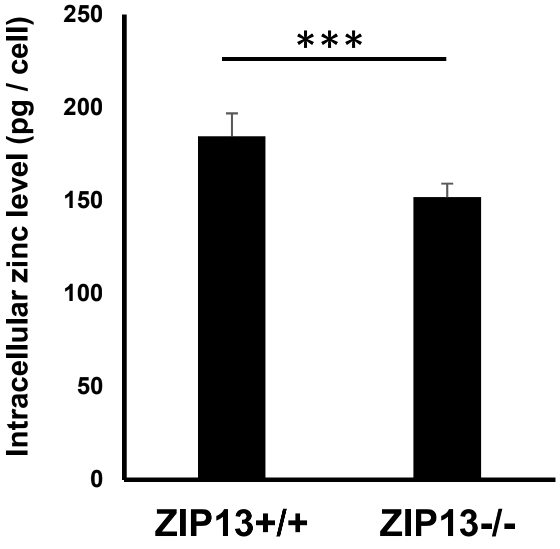

Figure S10. Measurement of the zinc level via inductively coupled plasma atomic emission spectroscopy. ***P < 0.005.

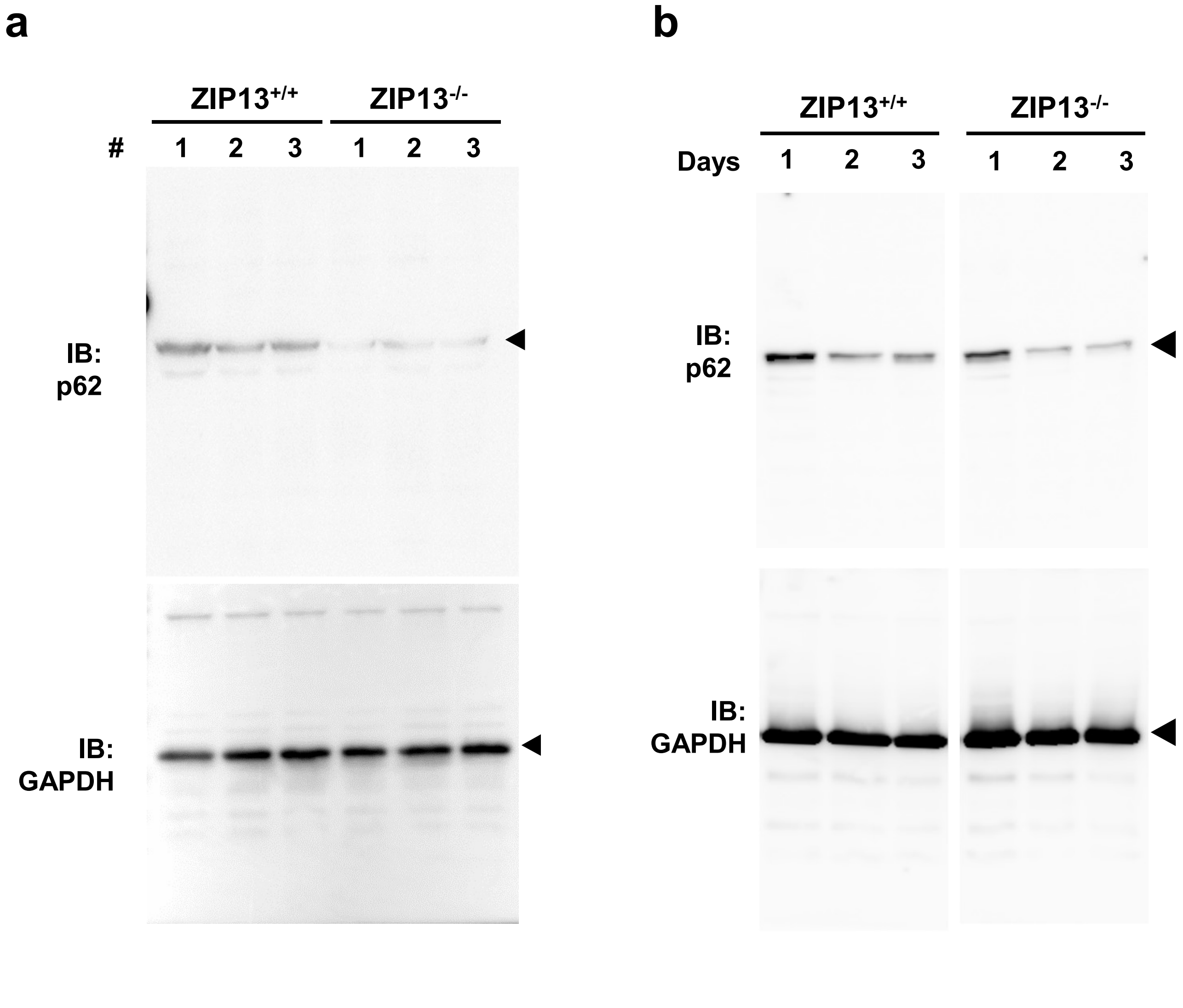

Figure S11. (a) Western blot analysis revealed downregulation of p62 proteins in the ZIP13$^{-/-}$ cell line. (b) Western blot analysis revealed that serum depletion significantly induces reduction of p62 protein in the ZIP13$^{+/+}$ cell line compared to the ZIP13$^{-/-}$ cell line.

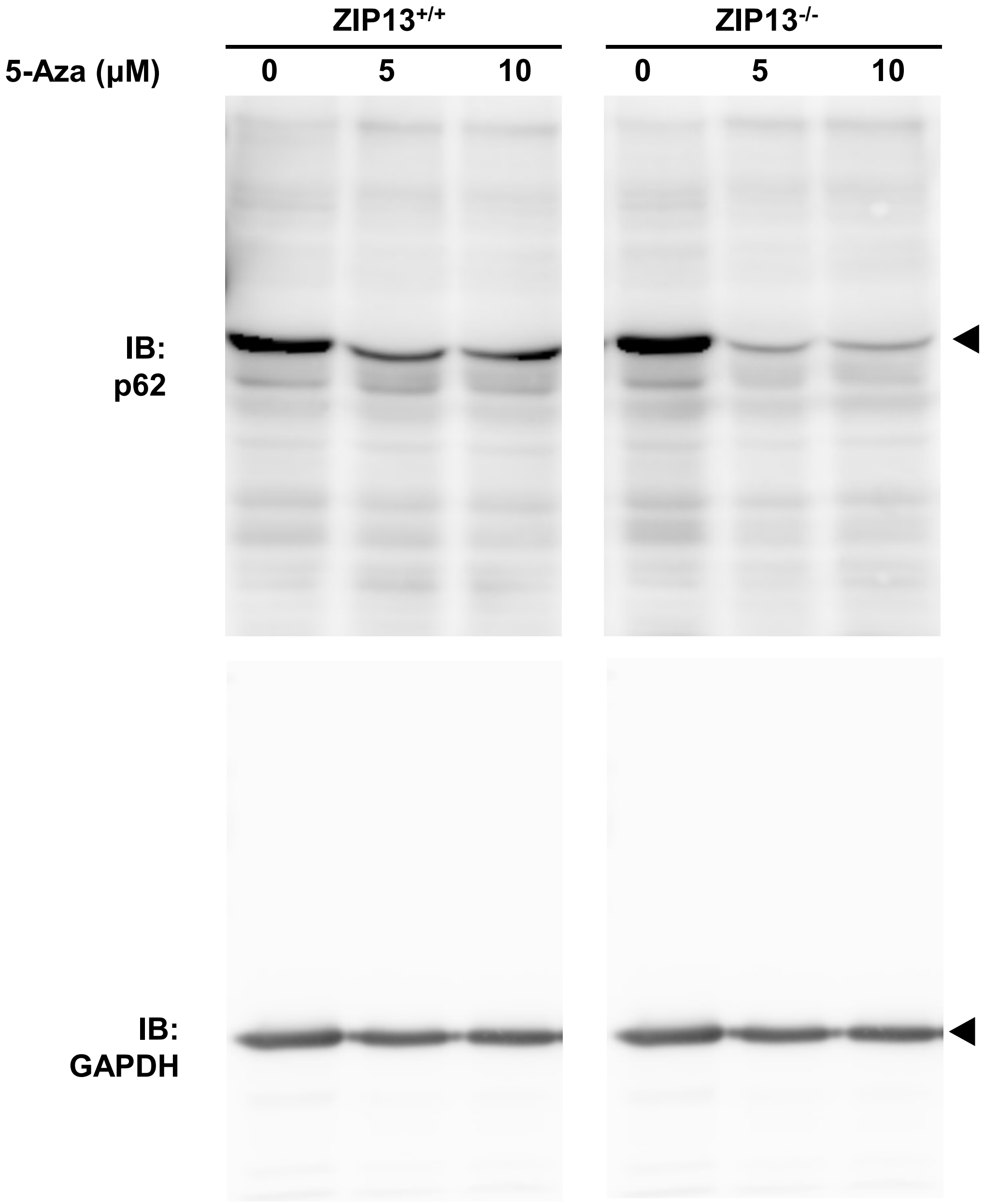

Figure S12. Western blot analysis revealed that 5-aza-2'-deoxycytidine (5-Aza) treatment for 24 h reduces p62 expression in the ZIP13$^{+/+}$ and ZIP13$^{-/-}$ cell lines.
